# A Plea for Caution: Huge Risks Associated with Lab-bred Flu

**DOI:** 10.3390/v4020276

**Published:** 2012-02-07

**Authors:** Viktor Müller

**Affiliations:** Research Group of Theoretical Biology and Ecology, Eötvös Loránd University and the Hungarian Academy of Sciences, Pázmány P. s. 1/C, 1117 Budapest, Hungary; Tel.: +36-1-3722500/ext.8798; Fax: +36-1-3812188; Email: mueller.viktor@gmail.com

**Keywords:** influenza, H5N1, pandemic, science policy

## Abstract

I wish to express concern about the maintenance of laboratory strains of H5N1 influenza viruses that might be adapted for transmission among humans.

A strain of highly pathogenic H5N1 flu has recently been modified to be transmissible among ferrets [1], which is a correlate of potential human-to-human transmission. Another group created a recombinant virus that carries the H5 hemagglutinin gene stitched into the genetic background of the 2009 pandemic flu virus and is also transmissible among ferrets [2] (Box 1). These studies have sparked a major controversy about the publication of the procedures used and the continuation of this research.

I believe that the studies done so far have yielded important insights: in particular, identifying a possible mutational pathway towards adaption to transmission among humans, demonstrating that adaptation to the new host does not necessarily reduce the high mortality associated with the parental strains, and showing that H5N1 “bird flu” can readily exchange genes with the currently circulating H1N1/09 human influenza virus. These results reinforce the threat posed by the strains of H5N1 flu that are currently circulating in birds, and might provide some chance for “early warning” if the emergence of the critical mutations can be monitored by global surveillance [1,2]. However, the same results clearly point out the risks associated with the continued maintenance of these modified strains in the lab.

I wish to join the sober voices that urge for a careful consideration of whether, or under what conditions, continued research on these strains should be carried out [3]. The potential risks must be weighed against the potential benefits, and these must be considered separately for both lab-modified strains, which seem to carry very different levels of benefits and risks.

In terms of benefits, it would be unrealistic to expect that continued research could avert a potential future pandemic of H5N1 flu. These viruses are evolving in hundreds of millions of birds, and this process cannot be controlled—we can only react once an outbreak has started. The greatest potential benefit would be a vaccine, developed with the help of the modified strains, which could protect against a natural outbreak of human-adapted pathogenic H5N1 in the future. However, even this optimal scenario has serious limitations. It is highly unlikely that an H5N1 vaccine would be stockpiled in the face of a vague risk for a natural outbreak and the uncertainty of the effectiveness of the vaccine when put to the real test. Should an outbreak happen, it will still take many months to years to get the vaccine to millions and billions of people: the first wave of the pandemic (which is typically the most virulent) will hit all the same. Furthermore, it is unclear whether a vaccine developed against the current lab-modified strains would be protective against a natural outbreak that emerges from the vast genomic pool of bird viruses (in particular, it might not be more protective than vaccines based on natural, less dangerous strains of H5N1 influenza virus [4]). Finally, any potential benefit of a vaccine will only be realized if a natural outbreak of pathogenic H5N1 influenza virus does occur, which seems to be a low-probability event. The current highly pathogenic strains of H5N1 influenza have been circulating among birds since at least 1996 [5], and have not been able to cause an outbreak in humans so far.

Furthermore, research on modified H5N1 influenza virus is by no means our “only hope” against the threat of an aggressive pandemic in the future. There are promising alternative fronts in the war against influenza viruses. While current seasonal vaccines have varying efficacy and must be re-designed each year, recent research indicates that a universal vaccine targeting conserved viral structures might be within reach now [6]. There is also no fundamental reason why broadly effective drugs could not be developed against influenza virus eventually, as has been done against other viral pathogens, e.g. HIV and HCV. However, research along these lines does not require the specifics of the modified influenza strains and can be carried out on much safer variants of the virus.

There might be a potential, though risky, use for the virus created by Kawaoka and colleagues, which seems to have retained the relatively benign nature of the 2009 H1N1 pandemic virus [2]. Should a natural outbreak of virulent H5N1 influenza virus occur among humans before broadly effective drugs or vaccines were available, this strain could be considered for a strategy of “fighting fire with fire”. Systematic global release of this virus might be able to elicit herd immunity against the H5 antigen, and protect against the devastation of the virulent pandemic at a cost comparable to that of the mild 2009 pandemic. This example illustrates that both modified strains, and any similar viruses in the future, need to be judged carefully and individually.

In terms of the risks, the worst-case scenario is a pandemic of virulent H5N1 influenza virus initiated by accidental lab release of the more aggressive strain [1]. Even if we have a vaccine by then, the first wave will hit and could kill millions, unless by pure chance the virulence of the virus attenuates very quickly. Influenza viruses have a proven track record of being able to kill millions within weeks [7], the current H5N1 viruses have shown high mortality both in birds and in the rare human cases [8], and the viruses modified in Fouchier’s lab have maintained their high virulence in the new ferret host [1]. The risk of eventual lab release is considerable (such incidents have happened in the past [9]) and increasing over time and with the number of labs that work with the virus. The potential of the modified strains to cause a human pandemic is unknown, but the whole point of the exercise was to mimic adaptation to humans: this risk is therefore quite real.

In all, I believe that the maintenance of ferret-adapted, virulent H5N1 virus [1] carries limited benefit conditional on a low probability event on one side, and enormous cost conditional on a relatively high probability event on the other. At a minimum, research should be restricted to a few selected labs with the highest levels of security (BSL-4) (as argued, e.g., in [10]) and each project should be justified with a considerable potential gain in warding off a future pandemic: mere curiosity and “basic research” in this case do not balance the risk of a lethal pandemic. Without clear and potentially highly rewarding goals (which have not been articulated), live stocks of the modified virus should not be maintained. In contrast, the recombinant virus that carries the H5 gene in the benign H1N1/2009 genetic background [2] poses much less risk and potentially more benefit, and might therefore be a better candidate for further research. Finally, such decisions should clearly be made above the level of funding bodies and local biosafety committees. Joining others [10], I suggest setting up an international panel of experts (e.g. under the WHO), dedicated to evaluating proposals that involve modifying the host specificity of any highly virulent non-human pathogen or increasing the virulence of a human pathogen. Funding bodies should require that any such research be evaluated and approved by the panel as a pre-requisite for funding.

The decision makers are facing enormous responsibility. Should a lab-released strain go on to kill millions, it will be hard to interpret this as anything other than as a tragic failure on the part of scientists to responsibly constrain their activities in the face of serious public health implications. We need to think ahead, and we need to raise our voices now.

## Box 1.

Two independent experiments have created viruses that carry genes from highly pathogenic strains of H5N1 “bird flu” and are transmissible by an airborne route among ferrets, the model organism for human-to-human transmission [11]. The two groups used different techniques. The study led by Ron Fouchier (Erasmus Medical Center, Rotterdam) worked with complete H5N1 virus, introduced mutations thought to enhance transmissibility in ferrets and humans, then transmitted the mutated virus serially among ferrets [1]. The result was a modified strain capable of airborne transmission among ferrets and highly efficient at killing the infected animals. The virus accumulated five key mutations during the adaptation process. In contrast, the study led by Yoshihiro Kawaoka (University of Tokyo and the University of Wisconsin-Madison) built on the 2009 H1N1 pandemic influenza A virus, and switched only the hemagglutinin gene (one of the major determinants of strain-specific immunity) to that of the virulent H5N1 virus [2]. The result was a mutant strain that was also capable of airborne transmission among ferrets but did not kill any of the infected animals. 
